# New Insights for Cellular and Molecular Mechanisms of Aging and Aging-Related Diseases: Herbal Medicine as Potential Therapeutic Approach

**DOI:** 10.1155/2019/4598167

**Published:** 2019-12-12

**Authors:** Yanfei Liu, Weiliang Weng, Rui Gao, Yue Liu

**Affiliations:** ^1^Graduate School of Beijing University of Chinese Medicine, Beijing 100029, China; ^2^Institute of Clinical Pharmacology of Xiyuan Hospital, China Academy of Chinese Medical Sciences, Beijing 100091, China; ^3^Cardiovascular Diseases Center, Xiyuan Hospital of China Academy of Chinese Medical Sciences, Beijing 100091, China

## Abstract

Aging is a progressive disease affecting around 900 million people worldwide, and in recent years, the mechanism of aging and aging-related diseases has been well studied. Treatments for aging-related diseases have also made progress. For the long-term treatment of aging-related diseases, herbal medicine is particularly suitable for drug discovery. In this review, we discuss cellular and molecular mechanisms of aging and aging-related diseases, including oxidative stress, inflammatory response, autophagy and exosome interactions, mitochondrial injury, and telomerase damage, and summarize commonly used herbals and compounds concerned with the development of aging-related diseases, including *Ginkgo biloba*, *ginseng*, *Panax notoginseng*, *Radix astragali*, *Lycium barbarum*, *Rhodiola rosea*, *Angelica sinensis*, *Ligusticum chuanxiong*, *resveratrol*, *curcumin*, and *flavonoids*. We also summarize key randomized controlled trials of herbal medicine for aging-related diseases during the past ten years. Adverse reactions of herbs were also described. It is expected to provide new insights for slowing aging and treating aging-related diseases with herbal medicine.

## 1. Introduction

**Figure 1:**
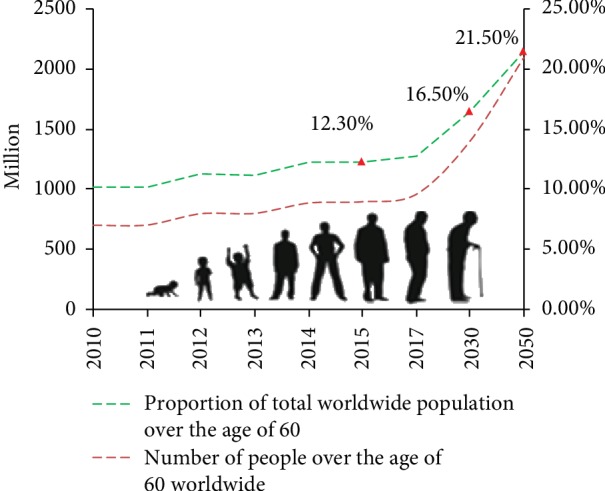
Epidemiological trends of aging worldwide.

**Figure 2:**
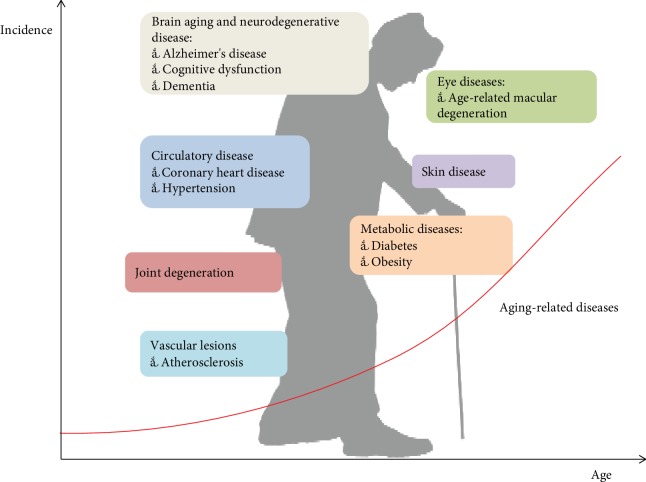
Main aging-related diseases with age and incidence.

Aging, which can be divided into pathological and physiological aging, is a complex biological process characterized by functional decline of tissues and organs, structural degeneration, and reduced adaptability and resistance, all of which contribute to an increase in morbidity and mortality caused by multiple chronic diseases [1, 2]. As fertility declines and life expectancy increases, the proportion of people aged 60 and older is increasing. According to the UNESA population division, approximately 900 million people are 60 years or older worldwide, and this will increase to 21.5% of the global population by 2050 [3] (see Figure 1). As aging progresses, it increases one's susceptibility to diseases associated with this process, such as vascular aging disorders [4–6], diabetes [7], muscle dysfunction [8, 9], macular degeneration [10], Alzheimer's disease (AD) [11, 12], skin diseases [13], and a series of other diseases [14–18] (see Figure 2). Aging-related diseases pose a serious threat to human health and reduce the quality of life among elderly people. In addition, it has become a global difficulty to clarify the mechanisms of aging, slow the process of aging, reduce the occurrence of aging-related diseases, and maintain that unfading appearance during the aging process.

Aging is a complex process with complicated mechanisms. At present, one of the accepted theories is related to oxidative stress [19–21]. In the process of aerobic metabolism, reactive oxygen species (ROS), including hydroxyl radicals, superoxide anions, and hydrogen peroxide (H_2_O_2_), can be produced in cells [22–24]. When ROS level exceeds the antioxidant capacity of cells, they react with lipids, proteins, and nucleic acids in cells, resulting in oxidation or peroxide formation. This leads to the destruction of the cell membrane structure, changes in permeability, and a cytotoxic reaction. High levels of ROS can directly damage DNA, proteins, and lipids in cells, causing a DNA damage response [25] and activation of p38MAPK for p16 upregulation. This leads to cell senescence and development of aging-related diseases [26]. DNA damage response also provides an appropriate trigger for the onset of telomere-induced senescence through the p53 pathway [25, 27, 28]. In addition to oxidative stress, various factors play a role in the aging process. Some reviews have reported the mechanisms of aging [26, 29–32]; however, only one of the mechanisms was examined; for example, some researchers [29, 30] described the role of miRNAs in aging while others [31, 32] placed an emphasis on autophagy.

In this review article, by mainly retrieving PubMed, here, we identify and critically analyzed nearly 10 years of published studies focusing on the mechanisms of aging and aging-related diseases, while summarizing some herbs and compounds that were more extensively used and studied for slowing aging. Compared with the latest published article describing the efficacy, mechanism, and safety of herbal medicine in slowing aging [33], this review is aimed at discussing the cellular and molecular mechanisms of aging from multiple perspectives, also emphasizing the interaction between exosome and autophagy in aging, and discussing age-related diseases and the progress of herbal medicine as potential therapeutic agents for aging and aging-related diseases. The adverse effects of herbs also get our attention in this review.

## 2. Cell Types Involved in Aging

### 2.1. Endothelial Cells

Endothelial cells are an essential part of the heart and vasculature [34]. They possess multiple functions through paracrine and endocrine actions, such as regulating vascular tension, maintaining blood circulation, and mediating inflammation, immune response, and neovascularization [35–37]. Endothelial dysfunction caused by endothelial cell senescence is closely linked to the development of aging. Several studies revealed that ROS and inflammation play a role in the apoptosis of endothelial cells [38–41]. Oxidative stress combined with thioredoxin-interacting protein (TXNIP) could activate NOD-like receptor family pyrin domain containing 3 (NLRP3) and inflammatory corpuscles during senescence of endothelial cells. In addition, the production of the proinflammatory cytokine, interleukin-1 (IL-1), which is induced by the activation of NLRP3 inflammatory corpuscles, could promote senescence of endothelial cells [42]. In recent years, it has been well established that autophagy and exosomes play significant roles in the course of a disease [43, 44]. Endothelial dysfunction and impaired autophagic activity are associated with age-related diseases [45]. Exosomes containing harbor miRNAs also participate in the regulation of endothelial function [46]. Studies demonstrated that miR-216a, a molecular component of miRNAs, could be induced during endothelial aging and play an important role in aging-related diseases by regulating autophagy-related genes, such as Beclin1 (BECN1) [47].

### 2.2. Stem Cells

Stem cells are pluripotent cells characterized as undifferentiated and immature with the ability to self-renew. Stem cell therapy is widely used in clinic, especially in cardiovascular regenerative medicine [48]. Under certain conditions, stem cells can be differentiated into various functional cells, with the potential function of regenerating various tissues and organs [49]. Changes in the cell cycle and a decline in the self-renewal ability of stem cells are closely related to aging. Although some changes in their function are intrinsic [50, 51], more external factors can lead to impairment in their function [52]. Studies have shown that the physiological levels of ROS could regulate the balance between self-renewal and stem cell differentiation [53, 54]. Nevertheless, oxidative stress due to high ROS levels could lead to DNA damage, shortening of telomeres [55], and the onset of premature aging markers, such as prelamin A, the lamin A. Nicotinamide adenine dinucleotide phosphate oxidase isoform 4 (Nox4) component of ROS could be localized to promyelocytic leukemia nuclear bodies (PML-NB) related to prelamin A, which could control the aging of stem cells [56]. Additionally, decline in self-renewal factors contributes to stem cell aging [57].

### 2.3. Vascular Smooth Muscle Cells

There are evidence suggesting that senescent vascular smooth muscle cells (VSMCs) have been observed in aging-related diseases, such as diabetes mellitus and atherosclerosis [58, 59], which indicate that senescent VSMCs contribute to aging. According to a study by Zhan et al. [60], VSMCs pretreated with the AMPK activator and mammalian target of rapamycin (mTOR) inhibitor could delay the replicative senescence of these cells. They revealed that the AMPK/TSC2/mTOR signaling pathway can regulate the replication and aging of VSMCs, which is mainly manifested as inhibition of the AMPK/TSC2/mTOR pathway which can inhibit the replication and aging of VSMCs. Another study showed that miR-34c-5p downregulation promoted VSM aging through a mechanism that might be mediated by the Bcl-2 modifying factor (BMF), which is a functional target of miR-34c-5p. LncRNAES3 was also found to act as a competing endogenous RNA (ceRNA) of miR-34c-5p to enhance BMF expression [61].

## 3. Molecules or Signal Transduction Pathways in Aging

### 3.1. Molecules in Aging

#### 3.1.1. MicroRNAs (miRNAs)

MicroRNAs (miRNAs, approximately 20-25 nucleotides) are a class of endogenous noncoding RNAs with regulatory functions found in eukaryotes. Recently, miRNAs were found to play an important role in aging [62–82] (see Table 1). According to a study by Du et al. [62], miR-17 extends the lifespan of transgenic mice by upregulating MKP and FoxO3 and downregulating mTOR and JNK through two targets, ADCY5 and IRS1. This study also found that ADCY5 or IRS1 can activate autophagy and inhibit cell aging and apoptosis. Dzakah et al. [63] demonstrated the role of miR-83 in modulating lifespan in Caenorhabditis elegans. Their study found that the deletion of miR-83 extended the lifespan of C. elegans and the expression of miR-83 decreased with age. The life-prolonging effect of miR-83 was achieved by high expression of the transcription factors, daf-16 and din-1. Lyu et al. [64] revealed that the regulation of transforming growth factor-*β* (TGF-*β*) signaling promotes senescence via miR-29-induced loss of H4K20me3. Their study found that miR-29 mediated the loss of suv4-20h2, downregulated H4K20me3 expression in mouse fibroblast senescent cells, and promoted cell senescence. Meanwhile, TGF-*β* accelerated cellular senescence by promoting the miR-29-mediated loss of H4K20me3. Fan et al. [65] observed the role of miR-1292 in cellular senescence of human adipose-derived mesenchymal stem cells (hADSCs). They found that FZD4 downregulation acted as a potential target of miR-1292, leading to overexpression of miR-1292, which promoted hADSC aging and osteogenic differentiation. This event was found to occur via the Wnt/*β*-catenin signaling pathway. Accumulating evidence suggest that miR-335-3p, which is neuron-enriched, is strongly linked to aging and age-related neurological diseases. Schilling et al. [66] found that statin-associated impairment of cognitive dysfunction is associated with PSD95 decrease, indicating that cholesterol levels are tightly linked to PSD95 levels. According to a study by Raihan et al. [67], overexpression of miR-335-3p, which could suppress cholesterol by inhibiting the expression of 3-hydroxy-3-methylglutaryl-CoA synthase-1 (HMGCS1) and 3-hydroxy-3-methylglutaryl-CoA reductase (HMGCR) in astrocyte, led to impaired cognitive function and memory. To add, the decrease in cholesterol levels was associated with the decrease in PSD95. When the miR-335-3p expression was reduced in the hippocampal brain of elderly patients, cognitive impairment and synaptic function could be restored in the aging process.

#### 3.1.2. Telomere

Telomeres, composed of the telomere DNA sequence and telomere protein, are nucleoprotein structures located at the end of chromosomes, which control the cell division cycle and maintain the genome's integrity [83]. Studies have shown that decreases in telomere attrition and telomerase activity are two of the main drivers of aging and age-associated damage that lead to cellular senescence [84]. The most well-established driver is the connection between adverse social conditions with DNA damage and accelerated telomere shortening [85, 86]. Epel et al. [87] used standardized questionnaires to assess the previous month's stress levels of 58 healthy premenopausal women. The control group included women with at least one healthy biological child, and the experimental group included the biological mother of a child with a chronic disease (*n* = 39). Mean telomere length and telomerase activity were measured to evaluate stress-induced changes. The results showed that stress in the experimental group was significantly higher than that in the control group. In addition, women in the experimental group had lower telomerase activity and shorter telomere length than those in the control group. These findings shed light on the cellular level of stress, which can affect one's health by modulating cell aging, possibly leading to the early onset of age-related diseases. Accumulated evidence indicates that DDR-related protein components are found in senescence-associated DNA damage foci (SDFs) [88]. Once ATM/ATR is activated, phosphorylation occurs in Chk1/Chk2, which further acts on effectors such as p53, leading to cell cycle arrest and failure to continue the cell cycle for a certain period of time, ultimately resulting in cell aging and even apoptosis [89, 90]. Further studies have also confirmed that telomere DNA shortening can induce ATM/ATR-mediated DDR and activate the downstream p53-p21 signal transduction pathway, leading to cell senescence [91].

#### 3.1.3. Sirtuins

Sirtuins containing seven different subtypes (SIRT1-SIRT7), which are members of NAD^+^ dependent histone deacetylase III, play an important role in cell stress resistance, energy metabolism, apoptosis, and aging [92]. Evidence exists that SIRT1 could deacetylate FOXO, block foxo-dependent transcription and the apoptotic pathway, and promote the survival of senescent cells. This occurs through an increase in SIRT1 expression with age, suggesting that Sirt1 is involved in longevity [93, 94]. SIRT 2 is closely related to age-related diseases, such as Alzheimer's disease (AD) and Parkinson's [95]. Studies have shown that inhibition of SIRT2 expression could delay the progression of these diseases. In addition, knockout of SIRT2 and SIRT5 could alleviate the neurodegenerative lesion induced by 1-methyl-4-phenyl-1,2,3,6-tetrahydropyridine (MPTP). The expression of SIRT2 was found to inhibit the dephosphorylation of FOXO3a and increase the level of Bim, leading to apoptosis and acceleration of the process of aging [96]. The mechanism by which SIRT5 deletion reduced apoptosis might be related to the reduction of SOD2 (manganese superoxide dismutase) expression [97]. SIRT3 has been reported to be associated with longevity. It can interact with FOXO3a to remove ROS and inhibit oxidative stress to prolong one's lifespan [98]. In the latest research by Zhang et al. [99], they found that by performing a whole-body knockout of “longevity gene” SIRT6 in nonhuman primates, they could obtain the world's first cynomolgus monkey model of longevity gene knockout, thereby revealing the new role of the SIRT6 gene in regulating embryonic development of primates. They could also elucidate the differences in aging and longevity regulation pathways between primates and rodents, laying an important foundation for research on the mechanisms of human development and aging and the treatment of related diseases [99]. SIRT7 could result in antiaging and prolong life by regulating the repair of the nonhomologous DNA damage to maintain the stable heredity of cells [100].

#### 3.1.4. Klotho Gene

The *Klotho* gene, located on human chromosome 13, contains five exons and exerts antiaging effects. Studies have confirmed that the decrease in *Klotho* expression with an increase in age leads to aging [101]. Ullah and Sun [102] found that lack of the *Klotho* gene reduced the activity of telomerase by modifying the expression of TERF1 and TERT, leading to apoptosis of pluripotent stem cells. Sustained exposure to Wnt accelerated cellular senescence both in vitro and in vivo [103]. However, studies revealed that the tissue and organs of *Klotho*-deficient animals could enhance the Wnt signaling pathway to cause cell senescence [103]. A few other studies showed that *Klotho* downregulation leads to premature aging of human fibroblasts, which might be achieved by regulating the insulin/IGF-1 pathway to upregulate p53 and p21 protein levels [104–106]. According to study by Gao et al. [107], *Klotho* deficiency could downregulate SIRT1, which reduce activities of AMP-activated protein kinase alpha (AMPK*α*) and endothelial nitric oxide synthase (eNOS), and upregulate NADPH oxidase activity, ultimately leading to aging-related aortic stiffness.

#### 3.1.5. p16, p53/p21

Cell cycle stagnation is the premise of aging [108]. Although cell aging involves a series of gene expression and cell morphological changes, which are not as simple as cycle stagnation, many experiments have confirmed that the increase in p16 or p53/p21 is enough to cause cell aging [89, 109–113]. In mouse embryonic fibroblasts, overexpression of miR-20a increased p16 and upregulated the transcriptional activity of INK4a/ARF, leading to cell senescence [114]. P53 is not only an initiator of cell aging but also a participant in antiaging. These effects of p53 are closely related to its involvement in the regulation of the mTOR pathway, which is closely related to autophagy. P53 can play an antiaging role by inhibiting the activity of mTOR and can also activate mTOR to inhibit the aging process [115]. Meanwhile, p53, through its downstream p53/p21/CDK2 signaling pathway, was found to result in cell cycle arrest and enter the aging state [111]. Studies have found that azithromycin might cause aging of VSMCs by activating the mTOR signaling pathway and increasing the expression of p53/p21/p16. When the activity of mTOR was inhibited, the autophagy level of proteins related to the mTOR signaling pathway increased, leading to a decrease in the expression of p53/p21/p16, thereby delaying the aging of VSMCs [116].

### 3.2. Signaling Pathway in Aging

#### 3.2.1. Mammalian Target of Rapamycin (mTOR) Pathway

mTOR, activated by growth factors and nutrients, inhibits autophagy and promotes protein synthesis. Over time, mTOR may promote cellular stress, such as protein aggregation, organelle dysfunction, and oxidative stress, which may lead to the accumulation of damage and cell function decline, ultimately promoting the occurrence of age-related diseases [117]. The classical pathway of mTOR is the PI3K/Akt/mTOR signaling pathway. Tan et al. [118] transfected human VSMCs with mTOR siRNA and scrambled siRNA and found that PI3K/Akt/mTOR plays a significant role in VSMC replication and aging, which might be related to the regulation of oxidative stress and telomere function. Additionally, mTOR activation induced stem cell depletion, which reduced tissue repair and aggravated tissue dysfunction. Experimental studies have also shown that by inhibiting the mTOR signaling pathway through gene knockout, rapamycin or dietary restriction can delay aging of various biological models, including yeast, worms, fruit flies, and mice [119].

#### 3.2.2. Nuclear Factor of Activated B-Cell (NF-*κ*B) Signaling Pathway

NF-*κ*B, activation of the transcription factor protein family, is involved in oxidative stress, immunity, inflammation, cell proliferation, apoptosis, and aging of gene transcription regulation. Studies have confirmed that NF-*κ*B has a highly conserved REL homologous domain consisting of 300 amino acids and that its protein family members include p50, p52, REL, REL-A, and REL-B [120]. The NF-*κ*B signaling pathway is activated by senescence-related inflammatory factors. Activated NF-*κ*B enters the nucleus and binds to DNA, thereby participating in cellular immune response [121]. Studies have confirmed that the occurrence of various senile degenerative diseases is closely related to the aging signaling pathway regulated by NF-*κ*B. Postmortem examination revealed an increase in NF-*κ*B activity in brain tissues of Alzheimer disease (AD) patients. In addition, the immunological activity of p65 was detected in neurons and glial cells adjacent to degenerative neurons and senile plaques [122]. The activation of NF-*κ*B is related to the deposition of *β*-amyloid (A*β*). Studies have found that A*β* deposition could activate NF-*κ*B in cultured neurons with the formation of NO products related to oxidative stress [123]. Autopsy studies found that the number of NF-*κ*B-positive dopaminergic neurons in the brain of patients with Parkinson's disease was 70 times higher than that of normal people, suggesting that the activation of NF-*κ*B is related to the pathological mechanism of Parkinson's disease [123, 124].

#### 3.2.3. Nuclear Factor-E2-Related Factor 2 (Nrf2) Signaling Pathway

Nrf2 is a key factor of antioxidant activity in cells. When oxidative stress occurs, Nrf2 is transferred to the nucleus to bind with the antioxidant response element (ARE) and regulates the expression of various antioxidant proteins and detoxification enzymes downstream, ultimately playing a role in endogenous protection [125]. Suh et al. [126] found that total Nrf2 protein and the amount of nuclear Nrf2 protein in rat liver cells significantly decline with an increase in aging. As age increases, the antioxidant capacity of ovarian cells decreases, and the imbalance between oxidation and antioxidants causes gradual apoptosis of ovarian cells, which is one of the important causes of ovarian aging. Studies have found that Nrf2 gene knockout can increase the ovary's sensitivity to toxic substances and accelerate the aging of ovaries in mice [127]. Chen et al. [128] found that the upregulation of Nrf2 expression could alleviate oxidative stress and DNA damage and inhibit the p53-p21 p16-rb signaling pathway, thereby slowing cell aging. Nrf2 can regulate mitochondrial biogenesis and kinetics to maintain muscle mass and function, and its deficiency with aging increasingly promotes age-related skeletal muscle mitochondrial dysfunction and muscle atrophy [129, 130]. Study also found that Nrf2 activation could inhibit age-related inflammatory responses and oxidative stress and delay the occurrence of aging and age-related diseases [131]. Activation of Nrf2 also improved learning and memory of aging mice administered with D-galactose (D-gal) [132].

#### 3.2.4. Wnt/*β*-Catenin Signaling Pathway

The Wnt/*β*-catenin signaling pathway is an evolutionarily, highly conserved signaling pathway with a wide range of biological functions. Studies found that this pathway plays an important regulatory role in cell aging and its activation could lead to senescence changes in mesenchymal stem cells [133]. Studies have also shown that activation of this pathway could lead to DNA damage response and increase the expression of the p53 protein, which might be one of the important mechanisms for stem cell senescence [134]. The p53/p21 pathway and DNA oxidative damage response have been confirmed to play an important role in the aging process of hematopoietic stem/progenitor cells caused by the Wnt/*β*-catenin signal pathway [135]. Skin aging is the most important external manifestation of human body aging, and the related components of WNT/*β*-catenin signal pathway are abnormally overexpressed in aged skin tissues [136]. The WNT/*β*-catenin signal pathway was found to be enhanced in the aging mouse model, and inhibition of the WNT/*β*-catenin signal pathway could reverse age-related skeletal muscle regeneration injury [137].

#### 3.2.5. Adenosine Monophosphate Protein Kinase (AMPK) Signaling Pathway

AMPK is a highly conserved cellular energy metabolism regulator that plays an important role in regulating cell growth, proliferation, survival, and energy metabolism [138]. AMPK is involved in the regulation of a series of senescence-related signaling pathways, such as SIRT1 and CRTC-1. Studies have shown that AMPK first enhanced the expression of niacinamide phosphoribose transferase and then increased the intracellular concentration of NAD^+^ to activate SIRT1, which then activates the downstream PGC-1, FoxO1, and FoxO3, ultimately interfering with the aging process [139]. Mair et al. [140] identified that CRTC-1 is the phosphorylation site of AMPK/AAK-2 with the nematode model, and AMPK/AAK-2 prevented its nuclear translocation via CRTC-1 phosphorylation, thereby inhibiting the transactivation of CREB transcriptional regulator crh-1 which extended the nematode's lifespan. AMPK activates p53 at certain phosphorylation sites and induces cell cycle arrest, leading to cell aging [141].

## 4. Aging-Related Diseases and Therapy

### 4.1. Vascular Aging

With an increase in age, the degeneration of vascular structure and function causes vascular sclerosis, which is called vascular aging. The main manifestations of vascular aging are increased arterial stiffness, pulse wave velocity, systolic blood pressure, and central venous pressure [142]. Vascular aging is a major risk factor for atherosclerosis and cardiovascular disease. Vascular aging mainly includes atherosclerosis and arteriosclerotic cardiovascular disease (ASCVD), such as coronary heart disease, hypertension, stroke, cognitive dysfunction, dementia, and peripheral vascular disease [143].

Studies have shown that decreased vasorin magnified the angiotensin II- (Ang II-) mediated increase in the TGF-*β*1 signaling cascade and caused vascular remodeling, thus leading to vascular aging [144, 145]. Increased Ang II with age led to activation of its downstream molecules MMP, McP-1, and TGF-*β*. This pathological change made the aortic wall of the elderly present a proinflammatory profile, which could promote atherosclerosis [146, 147]. Vascular endothelial cell senescence is one of the important pathological changes of vascular aging while oxidative stress is one of the main causes of endothelial senescence. eNOS has a significant effect on cardiovascular protection, and oxygenation should stimulate the decreased expression, resulting in a decrease in NO bioavailability, vascular diastolic dysfunction, and arteriosclerosis, ultimately promoting vascular aging [148]. Vascular endothelial cell aging is identified by ROS, the secretion of inflammatory cytokines, eNOS uncoupling, DNA damage, and telomere dysfunction, leading to obstacles in the structure and function of the cardiovascular system. It is also associated with coronary atherosclerotic heart disease [149, 150]. Studies have shown that atherosclerosis is associated with pathological thickening of vascular intima, loss of vascular smooth muscle cells, lipid deposition, and infiltration of macrophages [151]. Senescence was also found to accelerate atherosclerosis by inducing endoplasmic reticulum stress in VSMCs [152].

Complex functional impairment of cerebral microvessels and astrocytes may lead to neurovascular dysfunction and cognitive decline, which results in aging and age-related neurodegenerative diseases [153].

Early intervention of vascular aging can delay the occurrence of ASCVD and protect target organs. Presently, early intervention of vascular aging mainly includes lifestyle improvement and drug therapy. Caloric restriction and low-sodium diet combined with exercise can delay vascular aging. Meanwhile, active control of cardiovascular risk factors, such as hypertension, diabetes, and hyperlipidemia, can also prevent vascular aging. Drug therapy can target structural components of vascular aging, thus delaying development of aging. These mainly include antihypertensive drugs, statins, and hypoglycemic drugs. Antihypertensive drugs such as angiotensin-converting enzyme inhibitors (ACEI)/angiotensin-receptor antagonists (ARBs) have been shown to delay vascular aging due to their antifibrotic effects. Statins can not only regulate fat but also interfere with the process of vascular aging. Hypoglycemic drugs can increase the sensitivity of insulin, improve blood sugar, prevent the reconstruction of blood vessels, and inhibit inflammation of the tube wall.

### 4.2. Diabetes Mellitus

Diabetes is closely related to aging, and dysfunction of the pancreatic *β* cells plays an important role in the occurrence and development of diabetes. Aging of *β* cells in islets is mainly manifested as a decrease in the number of *β* cells and reduction in their secretion capacity. The mechanisms between islet cell failure in diabetes and aging are complex. Nonetheless, study found that the expression of autophagy signature proteins, LC3/Atg8 and Atg7, was decreased in aging islet cells. Similarly, the autophagy function of islets in aged rats was found to decrease [154]. Upregulation of P16ink4a/p19ARF expression, decrease in bmi-1 and EZH2 levels, and abnormal regulation of platelet-derived growth factor signals are important factors leading to a decline in the proliferation and insulin secretion of age-related *β* cells [155, 156]. The main interventions for diabetes include diet control, exercise, weight loss, and combination of hypoglycemic drugs.

### 4.3. Alzheimer's Disease

Alzheimer's disease (AD) is a neurodegenerative disease that occurs in old age and preold age. Brain aging is the basis and condition for the formation of neurodegenerative diseases. Alzheimer's disease is characterized by amyloid-*β* protein (A*β*) deposits that form plaques and by hyperphosphorylation of Tau protein that forms tangles of neurons (NFT). Abnormal mitochondria accumulate in neurons, leading to reduced ATP production, large release of oxygen-free radicals, the production of A*β*, and the intensification of Tau protein phosphorylation [157]. Mutations of PSEN 1 and PSEN 2 cause lysosomal dysfunction, and the presence of lysosomal dysfunction leads to a large number of autophagosomes generated by enhanced mitochondrial autophagy, leading to lysosomal overload and further aggravating brain injury [158]. Chronic activation of the NF-*κ*B pathway can cause the transcription of various inflammatory cytokines and promote glial cells to secrete inflammatory cytokines, leading to nerve cell injury and apoptosis [159, 160]. Currently, drugs used in the clinical treatment of AD are mainly noncompetitive N-methyl-D-aspartic acid receptor antagonists (such as memantine) and cholinesterase inhibitors (such as donepezil and galantamine).

### 4.4. Skin Aging

Skin aging, which is a part of the overall aging of the body, not only affects its appearance but also reduces its function as the body's barrier. This can lead to various diseases, such as depression and self-abasement. Tashiro et al. [161] cultured skin fibroblasts from women of different ages to study the relationship between autophagy and skin aging. They found that the autophagy degradation step was inhibited in skin fibroblasts of elderly donors, leading to the accumulation of autophagosomes. This suggests that the impairment of autophagy function in skin fibroblasts of elderly people may impact the skin's integrity and strength. Some researchers constructed a *Drosophila* model of skin aging and found that the increased expression of the autophagy marker, Atg7, was associated with skin aging [162]. Another study found that exosome miR-30a can regulate the apoptosis of epidermal cells, and its overexpression led to impaired epidermal differentiation by directly targeting AVEN (encodes a caspase inhibitor), IDH1 (encodes isocitrate dehydrogenase, an enzyme of cellular metabolism), and LOX (encodes lysyl oxidase, a regulator of the proliferation/differentiation balance of keratinocytes), inducing severe barrier dysfunction and skin aging [163]. Treatment for skin aging mainly includes oral antioxidant drugs, topical antiaging agents, and photoelectric and acoustic physical technology.

### 4.5. Aging-Related Macular Degeneration

Age-related macular degeneration (AMD) is one of the major causes of vision impairment in people older than 60 years of age. AMD can be divided into two types: dry AMD (atrophic), accounting for 85 to 90% of AMD cases and is a pattern of atrophy caused by the absence of retinal pigment epithelial cells and photoreceptor cells, and wet AMD (exudative, neovascular), which is caused by bleeding and exudation of neovascularization into the retina pigment epithelium (RPE) and into the sensory layer of the retina. Accumulating evidence suggests that the abnormal function of autophagy is related to the AMD formation. According to a study by Cai et al. [164], activation of mTOCR1 in aging RPE cells led to impaired lysosomal function and decreased autophagy in RPE cells. When the expression level of miR-29 is increased, the activity of mTORC1 is inhibited to enhance autophagy and remove protein aggregates to delay the occurrence of AMD. Another study found that SQSTM1/p62, a marker of autophagy injury, is deposited in the RPE along with the decrease in autophagy, which activates the inflammatory body, impairs protein clearance, and damages RPE cells, leading to AMD formation [165].

## 5. Herbal Medicines: Promising Therapeutic Agents for the Management of Aging and Aging-Related Diseases

**Figure 3:**
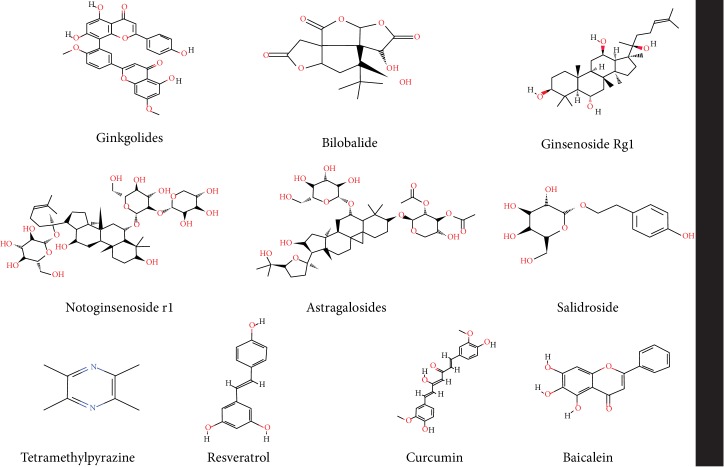
Chemical structural formula of the main active ingredients of herbs and compounds.

Studies had shown that many herbs had curative effect of slowing aging; selected herbs and compounds that were more extensively used and studied for review include Ginkgo biloba, ginseng, Panax notoginseng, Radix astragali, Lycium barbarum, Rhodiola rosea, Angelica sinensis, Ligusticum chuanxiong, resveratrol, curcumin, and flavonoids. The chemical structural formula of the main active ingredients of herbs and compounds was shown in Figure 3.

### 5.1. Herbs

#### 5.1.1. Ginkgo biloba (Yinxing)

*Ginkgo biloba extract* (EGb) has definite pharmacological effects of protecting the vascular endothelium, improving insulin resistance, and preventing atherosclerosis [166]. In addition, *EGb* exerts a good intervention in various age-related diseases, such as type 2 diabetes mellitus, dementia, cognitive impairment, and coronary heart disease [167]. The first international expert consensus regarding the clinical application of EGb for the treatment of dementia and moderate cognitive impairment was published in 2019 [168]. Dong et al. [169] pretreated senescent endothelial progenitor cells (EPCs) with 10, 25, and 50 mg/L of EGb and found that it could inhibit the senescence of EPCs and increase the activity of telomerase, especially at the concentration of 25 mg/L. The mechanism whereby EGb inhibited the aging of EPCs may be related to the activation of the PI3k/Akt signaling pathway. Zhou et al. [170] administered EGb-761 to aging mice at different doses of 20, 40, 80, and 100 mg/kg once every 3 days for 12 months and found that EGb could reduce ischemic injury and oxidative stress caused by ischemia in aging mice. Its mechanism might be related to the upregulation of protein phosphatase 2 (PP2A) and reduction in extracellular signal-regulated kinase (ERK) activation. Tian et al. [171] administered EGB to streptozotocin- (STZ-) induced diabetic ApoE^−/−^ mice at doses of 200 and 400 mg/kg/day for 12 weeks and found that EGb could regulate glucose and lipid metabolism, reduce arterial plaque, and upregulate autophagy to relieve endoplasmic reticulum stress (ERS). Its mechanisms might be related to the inhibition of ERS through the restoration of autophagy via the mTOR signaling pathway.

#### 5.1.2. Panax ginseng (Renshen)

Ginsenosides are the main active ingredients of *Panax ginseng*. Studies have shown that ginsenosides display plentiful pharmacological effects such as relieving fatigue, improving immunity, slowing aging, inhibiting metastasis of cancer cells, regulating blood glucose, and protecting liver and kidney functions [172].

Aging mice were intraperitoneally injected with the ginsenoside Rg1, at a dose of 20 mg/kg/day for 28 days continuously. Rg1 could retard testis senescence in mice via antioxidation and the downregulation of the p19/p53/p21 signaling pathway [173]. Zhou et al. [174] cultured aging Sca-1+ hematopoietic stem cells in ginsenoside for 6 h and found that ginsenoside could protect hematopoietic stem cells from aging. Its possible mechanisms of action might involve the regulation of the p16-Rb signaling pathway, the repair of worn telomeres, and maintenance of telomerase activity. Aging mice were fed an experimental diet based on AIN-93G containing 10 g/kg and 30 g/kg ginseng powder for 24 weeks continuously. The results suggested that long-term ginseng feeding could improve aging-related cognitive ability, which was achieved by regulating the cholinergic and antioxidant systems [175]. Other studies found that Rg1 could decrease oxidative stress and downregulate Akt/mTOR signaling to attenuate cognitive impairment in mice and senescence of neural stem cells induced by D-gal [176].

#### 5.1.3. Panax notoginseng (Sanqi)

*Panax notoginseng* contains the notoginseng saponins Rh1, Rh2, Rg1, Rg2, Rgb1, and others, with pharmacological actions such as antitumor activity, enhanced learning and memory, hemolysis, hemostasis, antiaging, and antifatigue [177, 178]. Zhou et al. [179] administered Panax notoginseng saponins (PNS) at 10, 30, and 60 mg/kg/day to natural aging mice and found that it could significantly and dose-dependently inhibit the apoptosis of myocardial cells in senescent rats by attenuating oxidative damage. Li et al. [180] pretreated aging H9c2 cells induced by D-gal with different concentrations of total saponins of Panax notoginseng (5, 25, and 50 g/mL) for 4 h. They found that the number of positive cells stained with galactosidase in the total saponins of the Panax notoginseng group was significantly reduced; SOD activity was found to significantly increase while MDA content and ROS fluorescence intensity were significantly decreased. Results suggest that PNS could resist aging of H9c2 cells induced by D-gal by improving their antioxidant capacity and reducing apoptosis.

#### 5.1.4. Radix astragali (Huangqi)

*Radix Astragalus* mainly contains astragalus polysaccharides, saponins, flavonoids, and other active components, which have various pharmacological actions such as antioxidation, antiaging, myocardium protection, and enhancement of immune function and hematopoietic function [181].

Ma et al. [182] used different doses (100, 200, 400, and 600 mg/kg) of *astragalus* extract for intervention in the animal model of sustained myocardial ischemia in vivo. They found that *Astragalus* can reduce myocardial injury and protect cardiac function, which are related to the reduction of oxidative damage and free radical generation. Ma et al. [182] also conducted in vitro experiments to interfere with the oxidative stress model of cardiac myocytes using *Astragalus membranaceus* at different concentrations (100, 200, 400, and 600 *μ*g/mL). They found that *Astragalus* could reduce the number of cell apoptosis by attenuating oxidative injury and arresting Ca^2+^ influx to block cell death. Li et al. [183] administered different doses (8, 16, and 32 mg/kg) of astragalosides via the intragastric route to the rat model with learning and memory impairment. They found that astragalosides could improve the learning and memory ability and ameliorate the neurodegenerative lesion of hippocampal CA1, which are related to the reduction of intracerebral amyloid precursor protein (APP) and a-secretase and *β*-secretase mRNA levels. Astragalus polysaccharides can also protect the mitochondria by scavenging ROS, inhibiting mitochondrial permeability transition (PT), and increasing antioxidant enzyme activity to improve aging in mice [184].

#### 5.1.5. Lycium barbarum (Gouqi)

*Lycium barbarum* has pharmacological actions such as regulating immunity, antitumor activity, nervous system function, liver protection, and slow aging process [185]. Hu et al. [186] administered different doses of Chinese wolfberry, via the intragastric route, to a mouse model of AD induced by the combination of AlCl_3_ and D-gal. They found that the quantity of horizontal and vertical movements increased while AChE and ChAT levels decreased significantly in mice. These events were related to the modulation of the mitochondrial pathway of apoptosis and the cholinergic system. Jeong et al. [187] used goji berry (150 and 300 mg/kg/day) to interfere with aging rats and found that goji berry could elevate the level of testosterone and reduce the expression of cell apoptosis activators, which are associated with its antioxidant action. Yu et al. [188] used *L. barbarum* to interfere with oxygen glucose deprivation and reoxygenation-induced injury of neurons. They found that *L. barbarum* inhibits oxygen glucose deprivation and reoxygenation-induced neuronal cell and autophagic cell death by activating the PI3K/Akt/mTOR pathway.

#### 5.1.6. Rhodiola rosea (Hongjingtian)

*Rhodiola rosea* contains alkaloids, flavonoids, glycosides, phenolic compounds, volatile oils, coumarins, steroids, and organic acids, plus small amounts of nonorganic elements, which could protect the heart and brain vessels by exhibiting antifibrosis, antioxidation, anti-inflammatory, antivirus, antiapoptosis, and antifatigue activities [189]. Zhou et al. [190] orally administered *R. rosea* (60 and 120 mg/kg daily) to an atherosclerosis rat model for 9 weeks continuously. The results showed that *R. rosea* could contribute to antiatherosclerosis via lowering blood lipids, antioxidant, and anti-inflammatory activities and by regulating endothelial function. Schriner et al. [191] demonstrated that *R. rosea* could prolong the lifespan of *Drosophila* by perturbing the silent information regulator 2 (SIR2) proteins, insulin and insulin-like growth factor signaling (IIS), and the target of rapamycin (TOR). Furthermore, *R. rosea* could prolong the life of silkworms by improving antioxidant capacity [192].

#### 5.1.7. Angelica sinensis (Danggui)

The active components of *Angelica sinensis* mainly include volatile oils (ligustilide, Angelica sinensis ketone), organic acids (ferulic acid, succinic acid, niacin, and azelaic acid), polysaccharides, and flavonoids (ferulic acid, succinic acid, niacin, anisolic acid, and azelaic acid) [193]. Zhang et al. [194, 195] orally administered Angelica polysaccharide (ASP, 200 mg/kg/) to aging mice induced by X-ray whole-body uniform irradiation. HSCs were then separated and purified after mice were sacrificed. The results showed that ASP could significantly reduce the positive rate of SA-*β*-gal staining and the proportion of G1 phase in the aging group of HSCs, reduce ROS production, downregulate p16 mRNA, and increase the ability of mixed colony formation and T-AOC. Cheng et al. [196] showed that ASP restored cognitive impairment caused by D-gal administration, promoted neural stem cell (NSC) proliferation, attenuated D-gal-induced NSC senescence, decreased the level of oxidative stress by enhancing antioxidative capacity, and decreased the levels of inflammatory cytokines of NSCs. These events slowed the aging speed by enhancing the antioxidant and anti-inflammatory capacity and downregulating the p53/p21 signaling pathway [197, 198].

#### 5.1.8. Ligusticum chuanxiong (Chuanxiong)

*Ligusticum chuanxiong* contains tetramethylpyrazine (TMP), ligustrazine, vanillin, emodin, ferulic acid, and other active ingredients which display various pharmacological actions in the cardiac and cerebrovascular system, nervous system, and respiratory system [199]. Chen et al. [200] demonstrated that TMP at different doses of 1, 3, and 10 mg/kg interfered with 6-ohda-induced Parkinson's disease in mice which confirmed that TMP protects against dopaminergic (DA) neurodegeneration and attenuates DA neuronal apoptosis by activating the PI3K/Akt/GSK3*β* signaling pathway. Wei and Wang [201] found that ligustrazine alleviated hypoxia-induced HUVEC cell injury, enhanced cell viability, and inhibited cell apoptosis, all of which are related to the upregulation of miR-135b and subsequent activation of JNK/SAPK and PI3K/AKT/mTOR pathways. These events promoted hypoxia-treated HUVEC cell growth. Another study has shown that TMP could inhibit the accumulation of senescent LepR^+^ mesenchymal stem/progenitor cells in bone marrow, reduce bone loss, and improve the metabolic microenvironment of aging mice via the AMPK-mTOR-Hif1a-VEGF pathway [202]. As a potential treatment, TMP could improve bone diseases related to human age and promote a healthy lifespan.

#### 5.1.9. Other Herbs

Hou et al. [203] selected aging, 24-month-old guinea pigs as the animal experimental models and fed them with a diet containing different doses (75, 100, or 150 mg/kg/day) of water-soluble extract components of *Salvia miltiorrhiza Bunge* for 28 days continuously. The study found a significant decrease in whole blood viscosity and improvement of blood viscosity and viscoelasticity at the dose of 150 mg/kg/day. Park et al. [204] gave old (20-month-old) specific pathogen-free male Sprague-Dawley rats with magnesium lithospermate B, extracted from Salvia at a dose of 2 or 8 mg/kg/day for 16 consecutive days. The results suggested that it reduces the renal damage of oxidative stress in old rats. After the researchers fed the fruit flies a full or dietary restriction diet supplemented with oregano-cranberry (OC) mixture, the study found that OC could extend the lifespan of fruit flies, especially females, while only OC supplementation at the young age interval increased reproduction in females [205, 206].

After 8 weeks of intraperitoneal injection of 100 mg/kg/d d-galactose to establish a rat model of aging with different doses of Ganoderma lucidum extract, it was found that Ganoderma lucidum could delay the progression of AD by regulating DNA methylation [207]. Lobo et al. [208] gave different concentrations (0.5-5.0 mg/mL) of the *Gynostemma pentaphyllum* extract to mouse dermal fibroblasts, which were placed under 8-watt ultraviolet C (UVC) light at a distance of 50 cm to induce oxidative stress. The results showed that *Gynostemma pentaphyllum* extract prolongs viability of mouse dermal fibroblasts damaged by UVC light-induced oxidative stress, especially at 4.5 mg/mL, and it suggested that *Gynostemma pentaphyllum* extract had potential therapeutic effect on dermal cell aging.

### 5.2. Compounds

#### 5.2.1. Resveratrol

Resveratrol is a natural polyphenol with anticardiovascular, anticancer, antibacterial, anti-inflammatory, antiaging, antineurodegenerative, and other pharmacological effects [209]. Wu et al. [210] used different doses (30 and 100 mg/kg/day) of resveratrol to intervene in mice with premature ovarian aging caused by chemotherapy. They found that resveratrol could improve premature ovarian aging caused by chemotherapy and ameliorate the renewal ability of oogonial stem cells by attenuating oxidative stress injury via Nrf2 activation.

Dehghani et al. [211] used resveratrol combined with calcitriol to intervene in D-gal-induced aging rats. This combination could protect the heart and its antioxidant status by modulating hemodynamic parameters and increasing the serum level of *Klotho*, respectively. Du et al. [212] used resveratrol (5, 10, and 50 *μ*M) to intervene in aging cells and found that it could improve cell activity and increase SOD level by regulating autophagy to achieve delayed aging. Amos et al. [213] intervened in damages to *Drosophila melanogaster* induced by 1-methyl-4-phenyl-1,2,3,6-tetrahydropyridine (MPTP) with resveratrol (0, 7.5, 15, 30, 60, and 120 mg/kg diet) and found that it could improve the survival rate, prolong the lifespan, and improve the behavioral defects of *D. melanogaster*; these effects were related to its anti-inflammatory and antioxidant activities.

Posadino et al. [214] treated HUVECs loaded with the ROS probe H2DCF-DA with different concentrations of RES (1–50 *μ*M), and the results showed that low concentrations of RES enhanced PKC activity, promoted DNA synthesis, and reduced apoptosis; high RES concentrations elicit an opposite effect. The results suggested that resveratrol had a biphasic concentration-dependent effect on endothelial cell survival, thus providing a guide for future investigation. Another study by Posadino et al. [215] showed low doses of resveratrol (0.5 *μ*M) effectively acting as an antioxidant agent by significantly reducing the roGFP oxidation state as compared with roGFP-infected control cells. With the increase of resveratrol dose, cell survival and metabolic activity decreased in parallel, suggesting that antioxidant and prooxidation effects were strongly related to dose. In addition, resveratrol was shown to increase skeletal muscle resistance to fatigue in aging mice for the alleviation of age-related skeletal muscle aging [216].

#### 5.2.2. Curcumin

Curcumin, the main component extracted from the rhizome of turmeric and zedoary, has various pharmacological actions, including antiaging, anti-inflammatory, and antioxidant actions [217–219].

Shailaja et al. [220] showed that curcumin could reduce the level of C-reactive protein (CRP) and enhance the level of malondialdehyde (MDA), which play a favorable role in slowing aging by inhibiting the expression of age-related inflammatory cytokines. By using concentrations of 1 *μ*M, 5 *μ*M, 10 *μ*M, and 20 *μ*M, Pirmoradi et al. [221] found the intervention effect of curcumin in rat adipose tissue-derived stem cells (rADSC) in vitro. Their results showed that curcumin could promote the proliferation of rADSC and reduce the senescence of adipose stem cells by promoting TERT gene expression. Hu et al. [222] revealed that curcumin could reduce extracellular matrix degradation and interstitial fibrosis induced by hypertension from modulating covalent histone modification and TIMP1 gene activation, thus protecting against hypertension-related vascular damage. Furthermore, curcumin could prolong the lifespan of *Drosophila* under heat stress conditions by increasing the antioxidant activity and mitigating the effect of heat shock responses [223]. Curcumin could also alleviate aging-related skeletal muscle mass loss and dysfunction [224].

#### 5.2.3. Flavonoids

Flavonoids are a kind of natural polyphenols, mainly including flavonoids, flavanols, flavonoids, anthocyanins, and isoflavones [225]. Studies had shown that flavonoids had definite efficacy in the treatment of age-related neurodegenerative diseases [226], cardiovascular diseases [227, 228], atherosclerosis [229], etc. Some progress has been made in the study of flavonoids in prolonging lifespan [230]. Hung et al. [231] injected 1-methyl-4-phenylpyridinium (MPP^+^, a Parkinsonian neurotoxin) into the brains of rats and randomly divided them into three groups which received different does (10, 30 mg/kg/day) of baicalein, a phenolic flavonoid for 7 days. The study found that baicalein could inhibit inflammatory activities and MPP^+^-induced apoptosis and autophagy in the nigrostriatal dopaminergic system of the rat brain. The results suggested that baicalein was of therapeutic significance in Parkinson's disease. Studies also showed that flavonoids could exert ameliorative antioxidant capacity and reduce A*β*-induced toxicity in Caenorhabditis elegans, thus prolonging lifespan of Caenorhabditis elegans [232, 233].

## 6. Adverse Effects of Herbal Medicine

Cianfrocca et al. [234] observed that a 49-year-old man received herbal therapy with Ginkgo biloba (40 mg, 3 times daily) for 2 weeks to improve his cognitive abilities, and the patient complained of two palpitations within a month. The 12-lead ECG had a normal morphology but showed sinus rhythm with frequent ventricular premature beats, and with the withdrawal of ginkgo biloba extract, electrocardiographic evidence of ventricular arrhythmias resolved. Erdle et al. [235] reported allergic reactions in two pediatric patients after inhaling and atomizing *American ginseng* powder, the former with urticaria and respiratory symptoms and the latter with recurrent allergic conjunctivitis, and there was evidence of sensitization to *American ginseng* on skin prick testing (SPT) (13 × 12 mm wheal). The researchers concluded that excessive oral administration of astragalus could cause allergy, headache, hypertension, or other symptoms; astragalus injection mainly caused fever, shock, and acute asthma [236]. Larramendi et al. [237] carried out a skin test of goji berry on 30 patients with plant food allergy and found that 24 patients showed positive results, which suggested that goji berries are potentially allergenic to people at high risk of food allergies. Chang et al. [238] reevaluated the postmarketing safety of depside salt injection (made from *Radix Salvia miltiorrhiza*) based on the real world and found that most common adverse drug reactions were headache, head distention, dizziness, facial flushing, skin itching, thrombocytopenia, and the reversibility of elevated aspartate transaminase. Chaudhari et al. [239] concluded that curcumin commonly used in dermatologic conditions may cause skin allergies, mainly manifested as contact urticaria.

The safety of drug use is one of the important contents of clinical pharmacology; herbal medicine has drawbacks in this respect. Further studies are needed to completely understand these widely used herbs or compounds and their efficacy in aging-related diseases.

## 7. Conclusion and Perspectives

**Figure 4:**
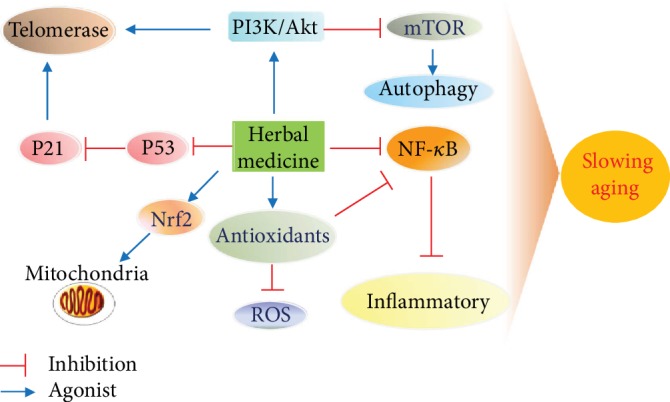
Possible mechanism of herbal medicines in slowing aging.

**Figure 5:**
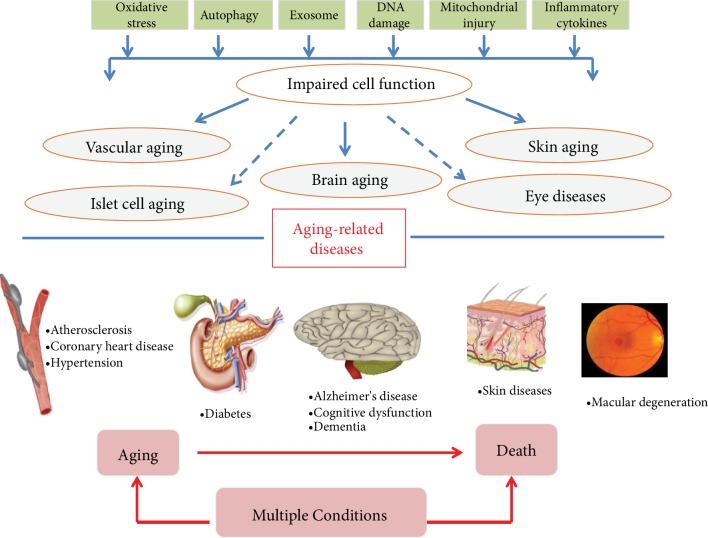
Possible mechanisms of aging and aging-related diseases.

Aging and aging-related diseases pose a serious threat to human health and reduce the quality of life of elderly people. Therefore, exploring the mechanisms of aging and against the occurrence of aging-related diseases is of great significance. In this paper, we discuss cellular and molecular mechanisms of aging and aging-related diseases, including oxidative stress, inflammatory response, autophagy and exosome interactions, mitochondrial injury, and telomerase damage (see Figure 4). We also discuss the possible mechanisms of age-related diseases (see Figure 5) and modern medical treatment for diseases related to aging. However, modern medicines result in many adverse reactions when used to treat aging-related diseases. Although drug therapy may improve the symptoms of early AD, they are not effective in patients with advanced AD and are associated with gastrointestinal toxicity. Intravitreal injection of antivascular endothelial growth factor is the most effective way to inhibit angiogenesis and control vascular leakage. However, intravitreal injection has many disadvantages which include risk of infection, the requirement of repeated treatment, and high cost. Most importantly, some patients still experience progressive visual impairment after treatment. Exploring the mechanisms of the multitargeted actions of herbal medicine will therefore help establish novel drugs for the treatment of aging-related diseases. In this review, we initially explored the possible mechanisms of herbal medicines in the treatment of aging and aging-related diseases (Table 2). Through in vivo and in vitro studies, various components of herbal medicine have been found to possess the ability to intervene in aging-related diseases by activating telomerase, increasing antioxidant capacity, reducing apoptosis and anti-inflammatory activities, and regulating aging-related pathways and exosomes. We also summarized the clinical randomized controlled trials (RCTs) of herbal medicine in the treatment of aging-related diseases (Table 3) [240–250]. These trials found that herbal medicine displays certain clinical efficacy in the treatment of age-related diseases such as type 2 diabetes, vascular dementia, AD, and atherosclerosis. A few clinical studies on AMD exist, but this disorder is considered to be related to the special technique used for intravitreal administration when treating macular lesions. Of note, as shown in Table 3, there are some adverse reactions in the clinical use of herbal medicines, including gastrointestinal discomfort, dry mouth, and abnormal alanine aminotransferase [245–247, 250]. Experimental studies had also found that there was a dose-response curve characterized by stimulation at a low dose and inhibition at a high dose. For example, the researchers used different concentrations of the drug to interfere with endothelial cells and found that cell survival rates decreased as the dose of the drug increased [214, 251–253]. This indicates that drugs have the effect of dose-dependent bidirectional regulation. When conducting study, attention should be paid not only to the dose-effect relationship but also to the optimal benefit concentration of drugs. Further analysis of the herbs mentioned in the article found that adverse reactions might occur with herbal treatment, such as palpitations, recurrent allergic conjunctivitis, urticaria and respiratory symptoms, fever, shock, and acute asthma [234–239]. Researchers should analyze the reasons for the adverse reactions and promote the standard and safe use of herbs.

In conclusion, high-quality RCTs should be carried out to observe the effectiveness and safety of herbal medicine in the treatment of aging and aging-related diseases. It is also important that the intervention of integrated traditional Chinese and western medicine be monitored in aging and aging-related diseases.

## Figures and Tables

**Table 1:** MicroRNAs involved in aging-related diseases.

miR type	Model	Function	Target gene	Reference
miR-17	H_2_O_2_ induced senescent cells	Inhibited mTOR and JNK activation	ADCY5, ISR1	[62]
miR-83	Caenorhabditis elegans	Inhibitory activity of miR-83	din-1, daf-16	[63]
miR-29	Senescent embryonic fibroblast cell	Mediated loss of H4K20me3 promotes senescence	Suv4-20h2	[64]
miR-1292	hADSCs	Accelerated hADSC senescence and restrained osteogenesis	FZD4	[65]
miR-335-3p	Male C57B/6J mice	Reduced cholesterol and impaired memory	Cholesterol	[66, 67]
miR-195	Neonatal mouse cardiomyocyte	Promote apoptosis, causing lipotoxic cardiomyopathy	SIRT1	[68]
miR-126	HUVECs	Regulate high-fat diet-induced endothelial permeability and apoptosis	TGF-*β*	[69]
miR-138	Aging participants	Regulating the memory function of the elderly	DCP1B	[70]
miR-451	Streptozotocin-induced diabetic mouse heart	Participated in cardiac fibrosis	TGF-*β*1	[71]
miR-34	Myocardial infarction (MI) in neonatal and adult mice	Its inhibition diminished cell apoptosis	Bcl2, cyclin D1, Sirt1	[72]
miR-146a	Human microvascular endothelial cells (HMVECs)	Ameliorates endothelial inflammation and the progression of atherosclerosis	Receptor-associated factor 6 (TRAF6)	[73]
miR-21	Human umbilical vein ECs	Promoting endothelial inflammation	PPAR*α*	[74]
miR-155	Human nasopharyngeal cancer and cervical cancer cells	Prevention of an age-induced deleterious decrease in autophagy	RHEB, RICTOR, RPS6KB2	[75]
miR-24	H9C2 cardiomyocytes	Attenuate cardiomyocyte apoptosis and myocardial injury	Keap1	[76]
miR-181	Apolipoprotein E-deficient mice	Dampen the inflammatory response in the endothelium	NF-*κ*B	[77]
miR-18a	Naturally aged mice	Regulation of extracellular matrix production during aging cardiomyopathy	CTGF, TSP-1	[78]
miR-377	Old skin tissues	Promotes fibroblast senescence	DNA methyltransferase 1 (DNMT1)	[79]
miR-9-5p	Human neuroblastoma cell line SH-SY5Y	Suppression in cell apoptosis, inflammation, and oxidative stress	SIRT1	[80]
miR-124	Normal human epidermal keratinocytes	Cause skin cell senescence	MEK1, cyclin E1	[81]
miR-15	Human dermal fibroblast	Counteracting senescence-associated mitochondrial dysfunction	SIRT4	[82]

**Table 2:** Preclinical studies of herbal medicine for aging-related diseases.

Active ingredients	Dosage	Administration	Model	Possible mechanism	Reference
In vitro studies					
EGb	10, 25, and 50 mg/L	Pretreatment for 24 h	EPCs cultured on fibronectin-coatedculture dishes	Activation of telomerase through the PI3k/Akt signaling pathway	[169]
Ginsenoside Rg1	10 *μ*mol/L	Cultured for 6 h	Aging Sca-1+ hematopoietic cells	Regulating the p16-Rb signaling pathway, repairing worn telomeres, and maintaining telomerase activity	[174]
PNS	5, 25, and 50 *μ*g/mL	Pretreatment for 4 h	D-Galactose induced aging H9c2 cells	Increase antioxidant capacity and reduce apoptosis	[180]
Astragalus membranaceus	100, 200, 400, and 600 *μ*g/mL	Pretreatment for 24 h	Cardiomyocyte model of oxidative stress	Attenuating the oxidative injury and arresting the influx of Ca^2+^ to block cell death	[182]
Lycium barbarum	15, 30, and 60 *μ*g/mL	Pretreatment for 24 h	Primary hippocampal neurons	Activating the PI3K/Akt/mTOR signaling pathway	[188]
Angelica sinensis	—	—	Aging hematopoietic stem cells	Increase in the length of telomere and the activity of telomerase, downregulation of the expression of P53 protein	[194]
Ligustrazine	50, 100, and 200 *μ*M	Pretreated for 24 h	Hypoxia-induced injury of HUVECs	Upregulation of miR-135b and subsequent activation of JNK/SAPK and PI3K/AKT/mTOR pathways	[201]
Gynostemma pentaphyllum extract	0.5-5.0 mg/mL	—	Mouse dermal fibroblasts induced oxidative stress	Reduce oxidative stress	[208]
Resveratrol	5, 10, and 50 *μ*M	Cultured for 24 h	H_2_O_2_ induced aging of HUVECs	Upregulation of autophagy	[212]
Curcumin	1, 5, 10, and 20 *μ*M	Treatment for 48 h	Rat adipose tissue-derived stem cells	Promoting TERT gene expression	[221]

In vivo studies					
EGb-761	20,40, 80, and 100 mg/kg	i.g. every 3 days, for 12 months	Aged mice (24 months) of middle cerebral artery occlusion	Upregulation of phosphatase PP2A and diminished extracellular signal-regulated kinase (ERK) activation	[170]
EGb	200, 400 mg/kg/day	i.g. 12 weeks	Streptozotocin-induced diabetic ApoE^−/−^ mice	Inhibiting endoplasmic reticulum stress via restoration of autophagy through the mTOR signaling pathway	[171]
Ginsenoside Rg1	20 mg/kg/day	i.p. 28 days	D-Galactose-induced aging mice	Antioxidation and downregulation of the p19/p53/p21 signaling pathway	[173]
Panax notoginseng saponins	10, 30, and 60 mg/kg/day	i.g. 6 months	Natural aging rats	Attenuating oxidative damage	[179]
Astragalus membranaceus	100, 200, 400, and 600 mg/kg	i.g. twice per day for 7 times	Rat model of persistent myocardial ischemia	Reducing oxidative damage and free radical generation	[182]
Astragalosides	8, 16, and 32 mg/kg	i.g. 14 days	Rats with learning and memory impairment	Downregulate the mRNA levels of APP and *β*-secretase, decrease expression of APP and A*β*_1–40_ in hippocampus	[183]
Astragalus polysaccharides	100, 200, and 300 mg/kg/d	i.g. 7 weeks	D-Galactose induced aging mice	Scavenging ROS, inhibiting mitochondrial PT, and increasing the activities of antioxidases	[184]
Lycium barbarum	0.5 or 2.0 g/kg	i.g. 4 weeks	A mouse model of AD induced by the combination of AlC_l3_ and D-galactose	Modulation of the mitochondrial pathway of apoptosis and the cholinergic system	[186]
Goji berry	150, 300 mg/kg	i.g. 6 weeks	Natural aging rats	Antioxidative stress	[187]
Rhodiola rosea	60, 120 mg/kg	i.g. 9 weeks	Abdominal aorta of atherosclerosis rats	Hypolipemic, antioxidant, and anti-inflammatory activities	[190]
Angelica polysaccharide	140 mg/kg	i.p. 27 days	Aging nestin-GFP mice induced by D-galactose	Enhancing the antioxidant and anti-inflammatory capacity, upregulation of p53/p21 signaling pathway	[196]
Tetramethylpyrazine	1, 3, and 10 mg/kg	i.p. 7 or 14 days	6-OHDA-induced Parkinson's disease mice	Activation of PI3K/Akt/GSK3*β* signaling pathway	[200]
Resveratrol	30, 100 mg/kg/d	i.g. 2 weeks	Mice with chemotherapy-induced ovarian aging	Attenuating oxidative stress injury by activating Nrf2	[210]
Curcumin	100, 200, and 400 mg/kg/d	i.g. 6 months	Natural aging rats	Suppressing age-related changes in inflammatory indices	[220]
Baicalein	10, 30 mg/kg/day	i.p. 7 days	MPP^+^-induced Parkinson's disease mice	Inhibit inflammatory activities and MPP^+^-induced apoptosis and autophagy	[231]

Abbreviations: EGb: Ginkgo biloba extract; EPCs: endothelial progenitor cells; HUVECs: human umbilical endothelial vein cells; i.g.: intragastric gavage; i.p.: intraperitoneally injected.

**Table 3:** Published randomized controlled trials of herbal medicines for the treatment of aging-related diseases in humans.

Number	Authors (year)	Targets	Conditions	Age (years)	Name of herb or formula	Dose/duration	Groups	Main outcomes	Adverse reactions
(1)	Liu et al. (2007) [240]	*n* = 66	Aging vascular dementia	≥55	Kangxin capsule (*Fructus lycii*, *Herba epimedii*, *Radix paeoniae alba*, *Radix Salvia miltiorrhiza*, *Fructus crataegi*, *Radix astragali*, *etc*.)	0.9 g once and three times per day, for 1 month	I: compound C: piracetam	CD4, CD4, CD8^−1^ ↑ (*P* < 0.05) HIS index, GDS, ET, E_2_·T^−1^ ↓ (*P* < 0.05)	No adverse reactions were observed
(2)	Zhao et al. (2018) [241]	*n* = 140	Type 2 diabetes mellitus	50-75	Ginkgo leaf tablets Liuwei Dihuang pills	2 Ginkgo leaf tablets and 8 Liuwei Dihuang pills, 3 times a day, for 36 months	I: compound C: placebos	Plasma CML, 8-IsoP levels ↓ (*P* < 0.05) FBG, PBG, BP, HbA1c, TC, TG, LDL-C, HDL-C (*P* > 0.05)	Drug reaction
(3)	Kwok et al. (2014) [242]	*n* = 165	Atherosclerosis in postmenopausal	56.0 ± 3.8	DG capsules (Danshen and ginseng)	Two capsules daily, for 12 months	I: compound C: placebos	TC, LDL-C carotid IMT ↓ (*P* < 0.05) BP, BMI, Glu (*P* > 0.05)	No adverse reactions were observed
(4)	Dingzhu et al. (2015) [243]	*n* = 156	Carotid atherosclerosis	57.7 ± 4.4	Shoushen granule (*Radix Polygoni multiflori*, *Fructus lycii*, *Crataegus*, and *Radix notoginseng*)	1 tablet once daily for 24 weeks	I: compound C: pravastatin	baPWV, IMTEp, AI, PWV*β* ↓ (*P* < 0.05)	Not reported
(5)	Lv et al. (2016) [244]	*n* = 69	Type 2 diabetes mellitus	50-80	Naoxintong (*Radix astragali*, *Angelica sinensis*, *Radix paeoniae rubra*, and *Ligusticum wallichii*)	1.2 g per day for 3 months	I: compound C: blank control	HbA1c ↓ (*P* < 0.05) Proliferative effects, migration ability, antiapoptotic function of HUVECs ↑ (*P* < 0.05) TC, TG, LDL-C, HDL-C (*P* > 0.05)	Not reported
(6)	Akhondzadeh et al. (2003) [245]	*n* = 42	Alzheimer's disease	65-80	Salvia officinalis extract	60 drops daily for 16 weeks	I: compound C: placebos	ADAS-cog, CDR-SB ↓ (*P* < 0.05)	Vomiting, wheezing, nausea
(7)	Akhondzade et al. (2010) [246]	*n* = 46	Alzheimer's disease	72.65 ± 3.89	Saffron	15 mg twice per day, for 16 weeks	I: compound C: placebos	ADAS-cog, CDR-SB ↓ (*P* < 0.05)	Dry mouth
(8)	Jia et al. (2014) [247]	*n* = 325	Vascular dementia	64.9 ± 9.1	SaiLuoTong (Ginkgo biloba, ginsenosides, saffron)	360/240 mg daily, for 52 weeks	I: compound C: placebos	VaD Assessment Scale—cognitive subscale scores (*P* < 0.05, 26 weeks)	Mild gastrointestinal intolerance, abnormal alanine aminotransferase, dreaminess
(9)	Tajadini et al. (2015) [248]	*n* = 44	Alzheimer's disease	>50	Davaie Loban	500 mg, three times daily, for 3 months	I: compound C: placebos	ADAS-cog, CDR-SB ↓ (*P* < 0.05)	Without any adverse drug reaction
(10)	Uno et al. (2005) [249]	*n* = 115	Type 2 diabetes	64 ± 1	Goshajinkigan	7.5 g daily for 1 month	I: combined compound and OHAs C: OHAs	HOMA-R, FBG TC, TG ↓ (*P* < 0.05) HbA1c (*P* > 0.05)	No adverse reactions were observed
(11)	Cho et al. (2009) [250]	*n* = 82	Healthy female	53.6 ± 7.4	Red ginseng root extract mixed with Torilus fructus and Corni fructus	3 g daily for 24 weeks	I: compound C: placebos	Facial wrinkles ↓ Type I procollagen gene, protein expression ↑	Gastrointestinal discomfort

Abbreviations: GDS: Geriatric Depression Scale; HIS: Hachinski Ischemia Scale; ET: endothelin; E_2_·T^−1^: estradiol (E_2_)·testosterone (T)^−1^; CML: carboxymethyl lysine; 8-IsoP: 8-isoprostane; FBG: fasting blood glucose; PBG: postprandial blood glucose; HbA1c: glycosylated hemoglobin; TC: total cholesterol; TG: triglyceride; HDL: high-density lipoprotein; LDL: low-density lipoprotein; IMT: intima-media thickness; GLU: glucose; Ep: pressure-strain elastic modulus; Ac: arterial compliance; AI: augmentation index; PWV*β*: pulse wave velocity *β*; HUVECs: human umbilical vein endothelial cells; ADAS-cog: cognitive subscale of Alzheimer's Disease Assessment Scale; CDR: Clinical Dementia Rating; OHAs: oral hypoglycemic agents.

## Abbreviations

ACEI: Angiotensin-converting enzyme inhibitors
AChE: Acetylcholinesterase
ACS: Acute coronary syndrome
AD: Alzheimer's disease
ADCY5: Adenylate cyclase 5
Akt: Protein kinase B
AMD: Age-related macular degeneration
AMPK: Adenosine 5′-monophosphate-activated protein kinase
AMPK*α*: AMP-activated protein kinase alpha
ARBs: Angiotensin-receptor antagonists
ARF: Auxin response factor
BECN1: Beclin1
ChAT: Choline acetyltransferase
CRTC-1: CREB-regulated transcription coactivator1
EGb: Ginkgo biloba extract
eNOS: Endothelial nitric oxide synthase
EPCs: Endothelial progenitor cells
EZH2: Enhancer of zeste homolog 2
FOXO: Forkhead box class
FZD4: Frizzled class receptor 4
H_2_O_2_: Hydrogen peroxide
hADSCs: Human adipose-derived mesenchymal stem cells
HMGCR: 3-Hydroxy-3-methylglutaryl-CoA reductase
HMGCS1: 3-Hydroxy-3-methylglutaryl-CoA synthase-1
IL-1: Interleukin-1
INK4: Inhibitors of cyclin-dependent kinase 4
ISR1: Insulin receptor substrate-1
miRNA: MicroRNA
MPTP: 1-Methyl-4-phenyl-1,2,3,6-tetrahydropyridine
mTOR: Mammalian target of rapamycin
NAD^+^: Nicotinamide adenine dinucleotide
NF-*κ*B: Nuclear factor of activated B-cells
NLRP3: NOD-like receptor family pyrin domain containing 3
NO: Nitric oxide
Nox4: Nicotinamide adenine dinucleotide phosphate oxidase isoform 4
Nrf2: Nuclear factor-E2-related factor 2
NSC: Neural stem cells
PML-NB: Promyelocytic leukemia nuclear bodies
PNS: Panax notoginseng saponins
PSEN: Presenilin
ROS: Reactive oxygen species
SIR1: Sirtuins 1
SOD2: Manganese superoxide dismutase
TGF-*β*: Transforming growth factor-*β*
TRAF6: Receptor-associated factor 6
TSC2: Tuberous sclerosis complex 2
TXNIP: Thioredoxin-interacting protein
VSMCs: Vascular smooth muscle cells.
